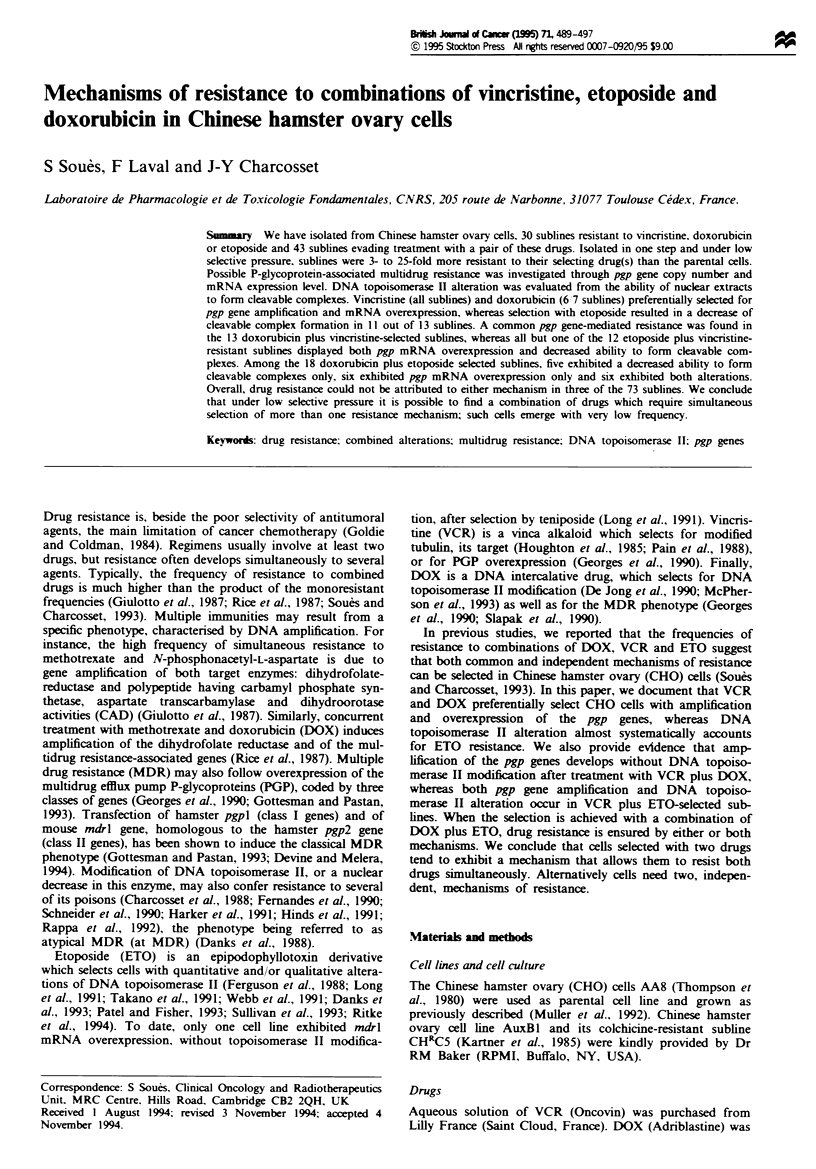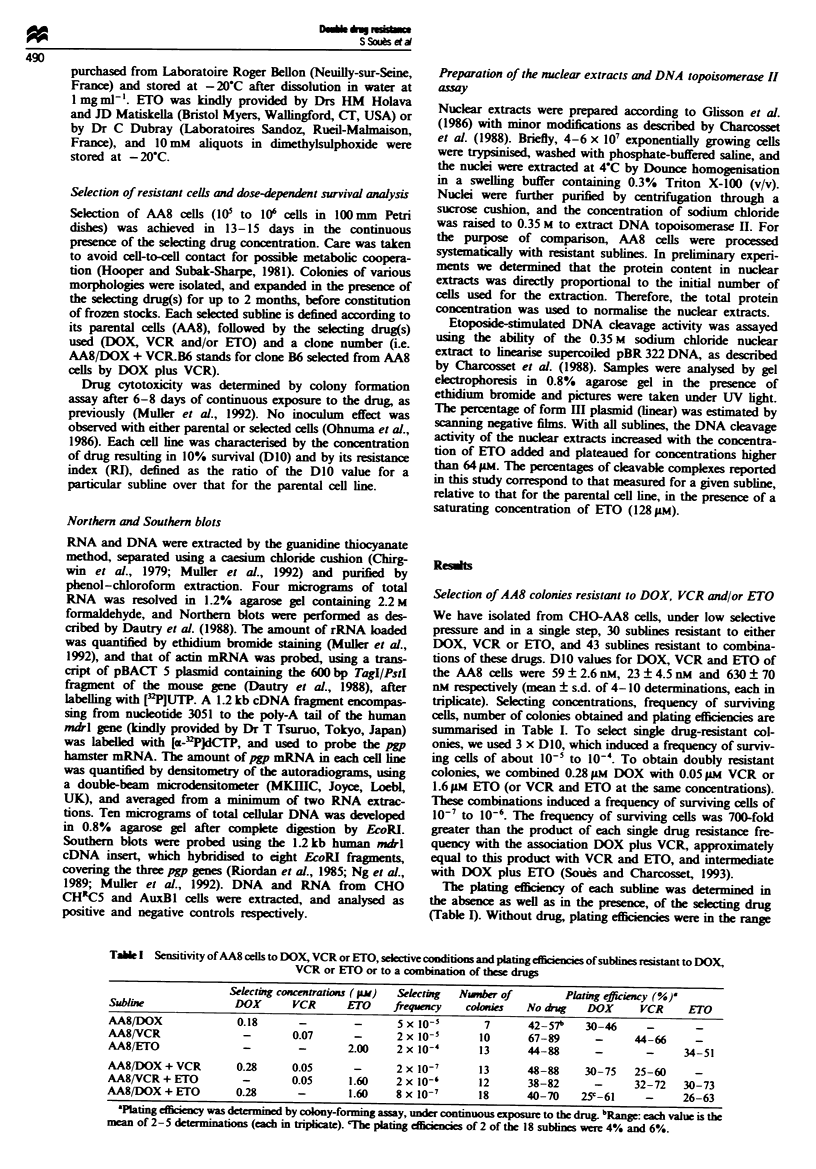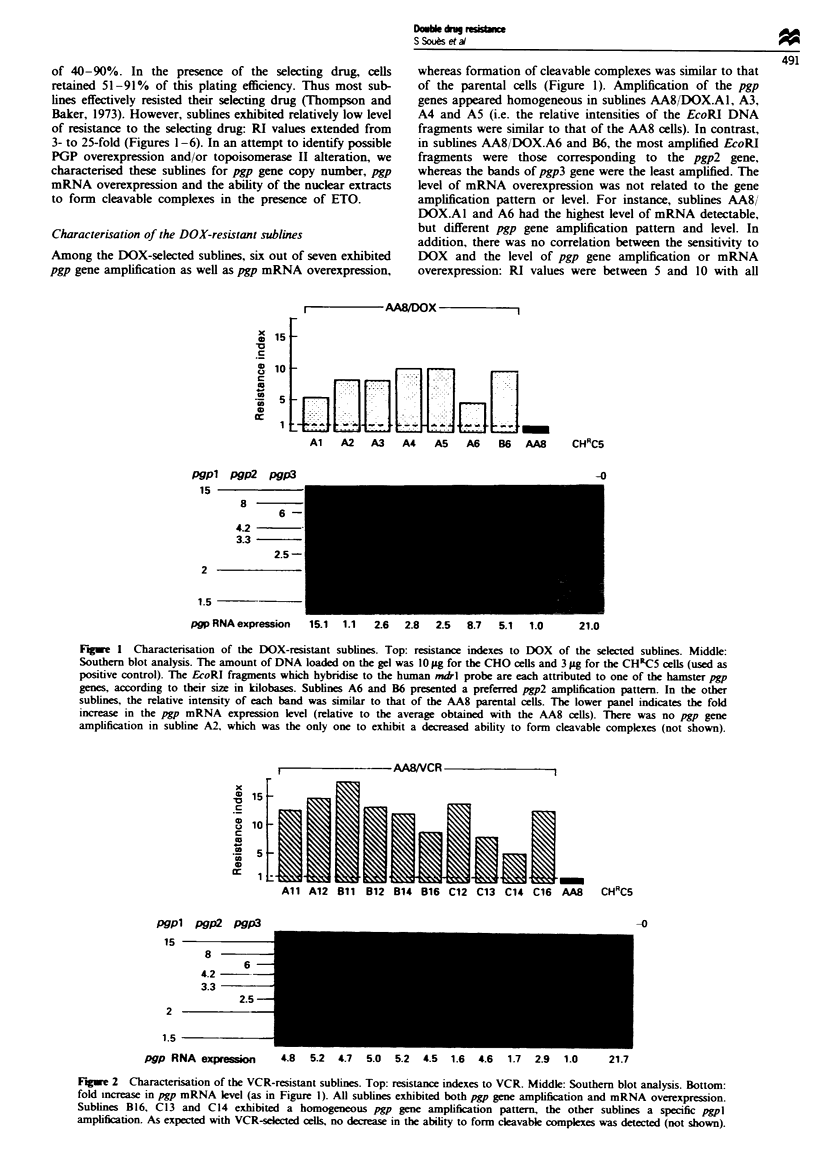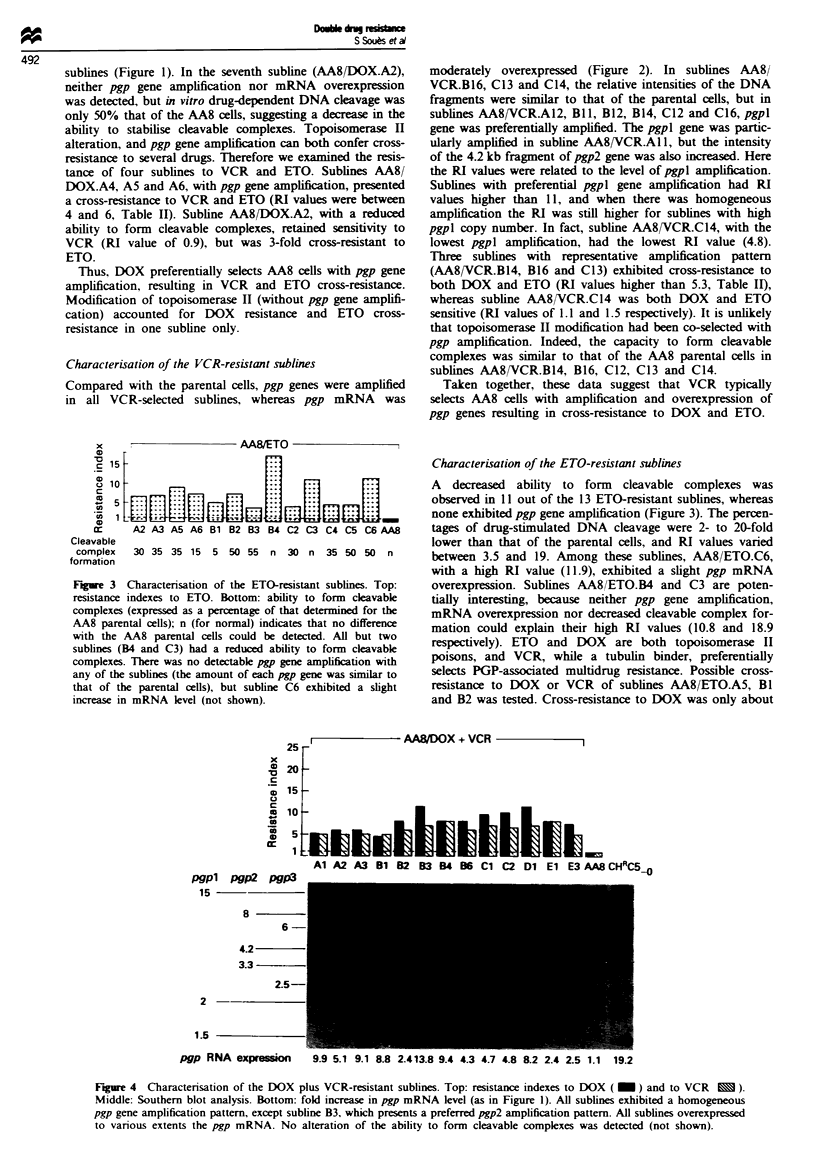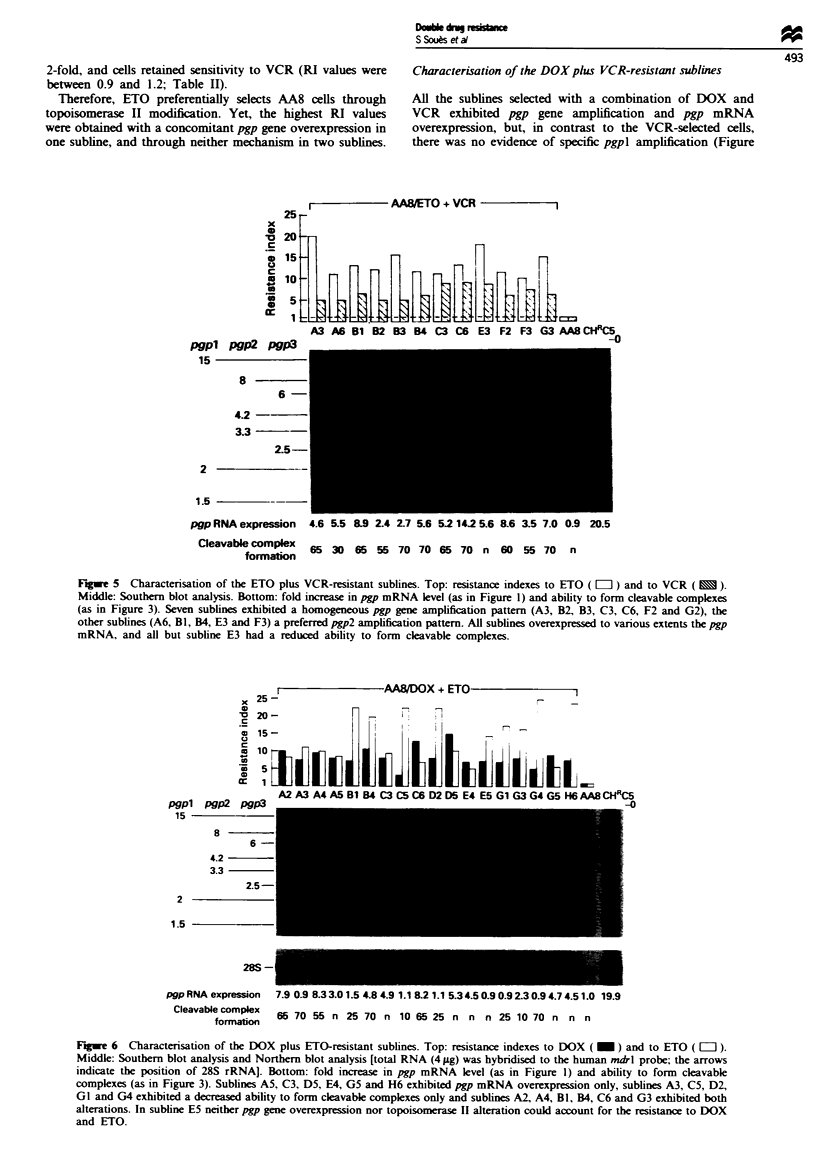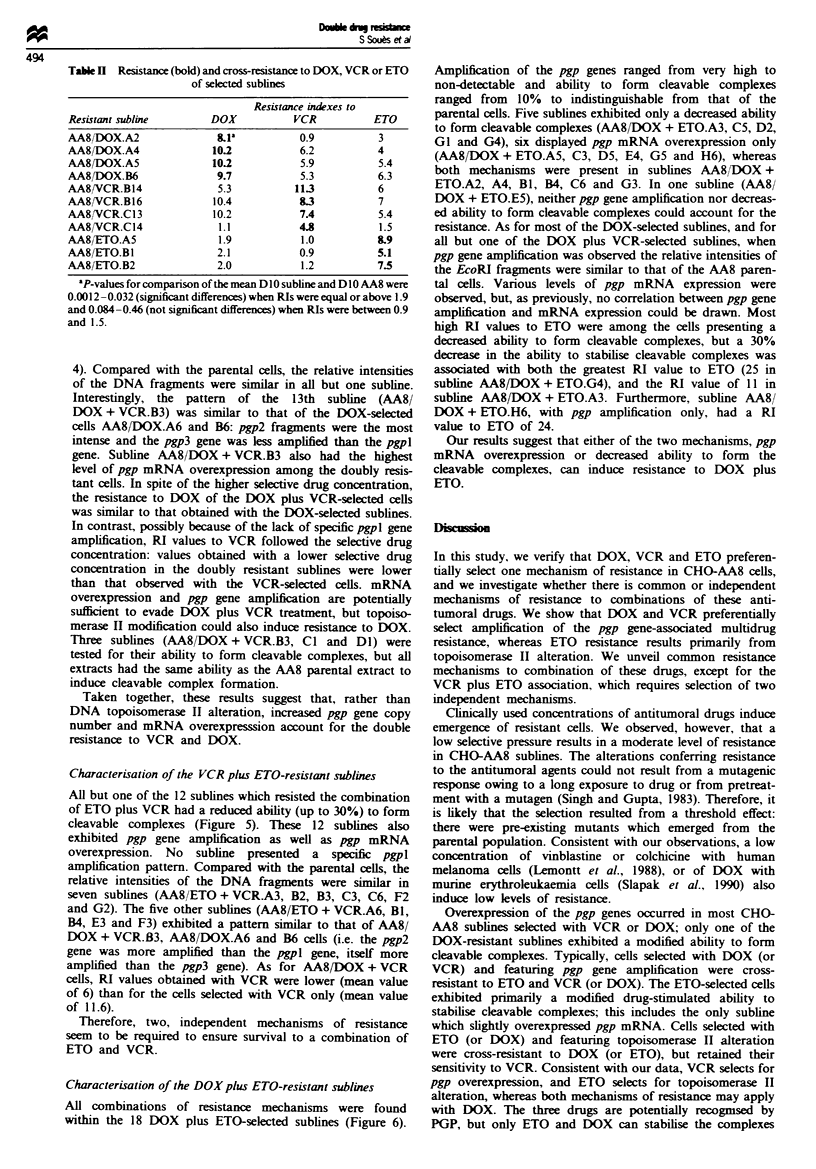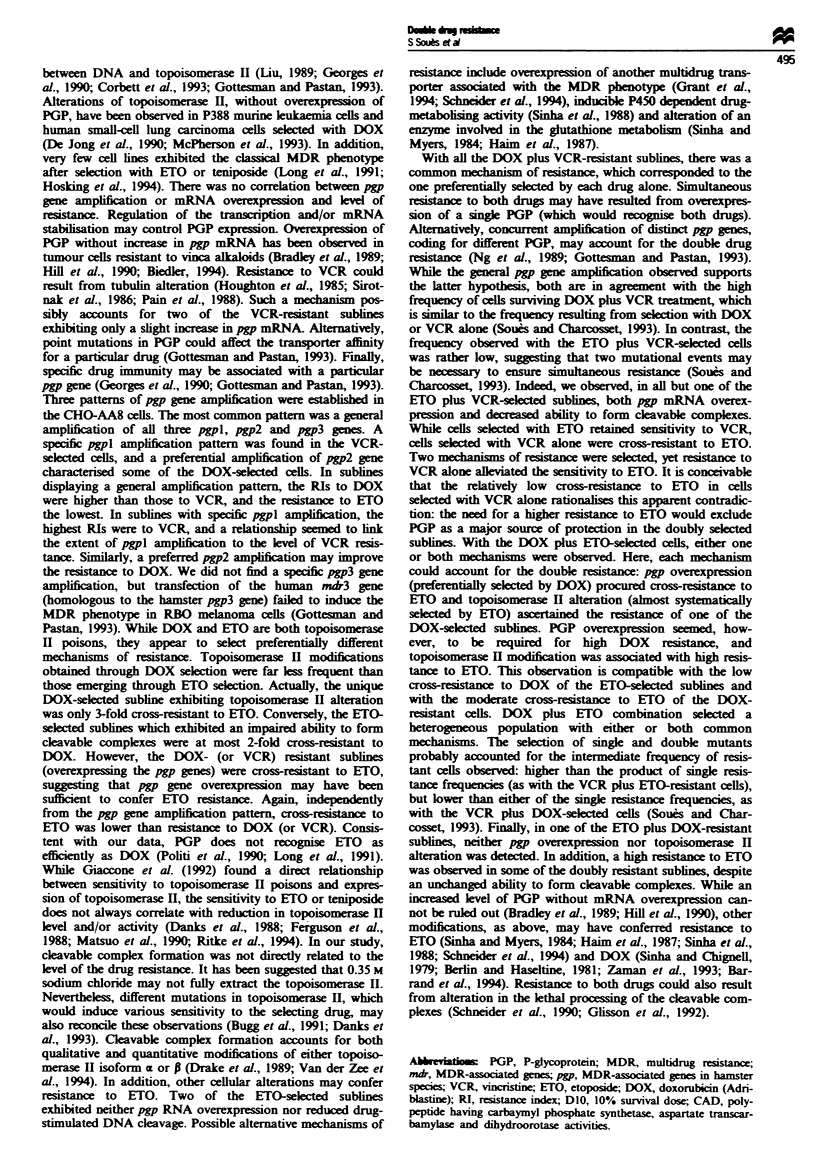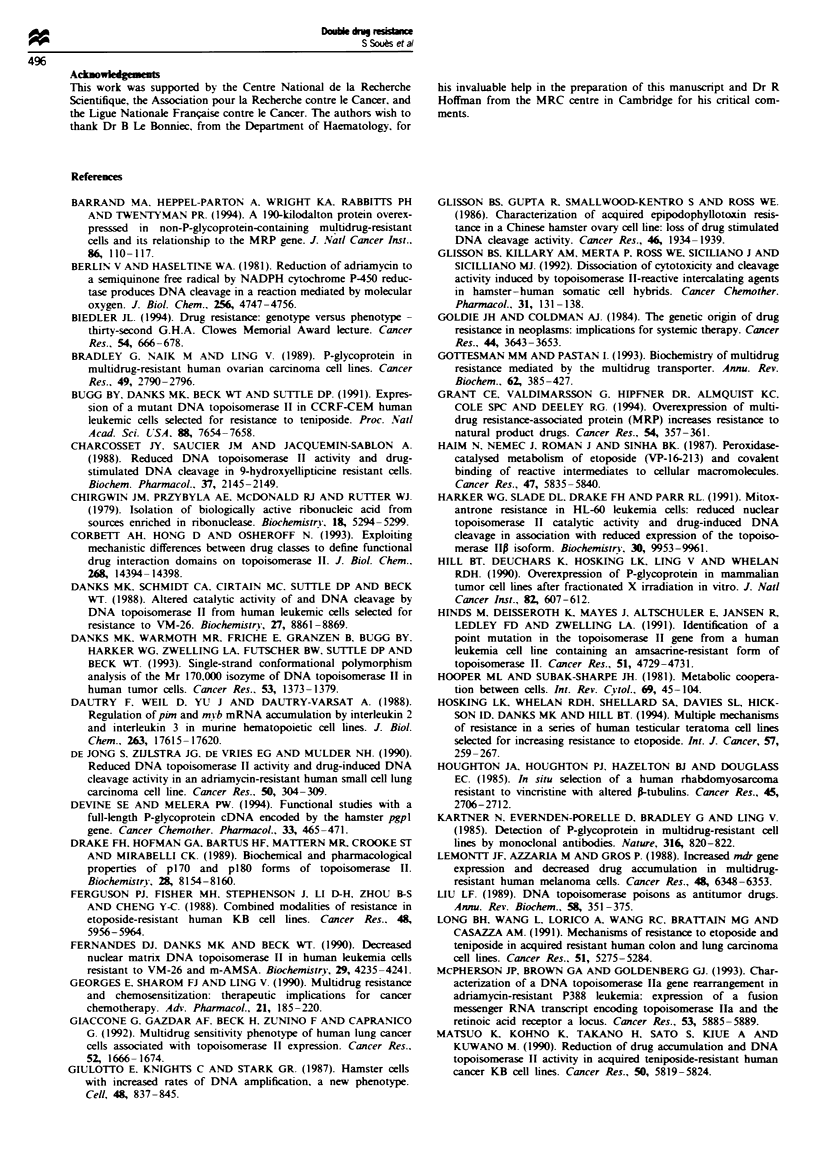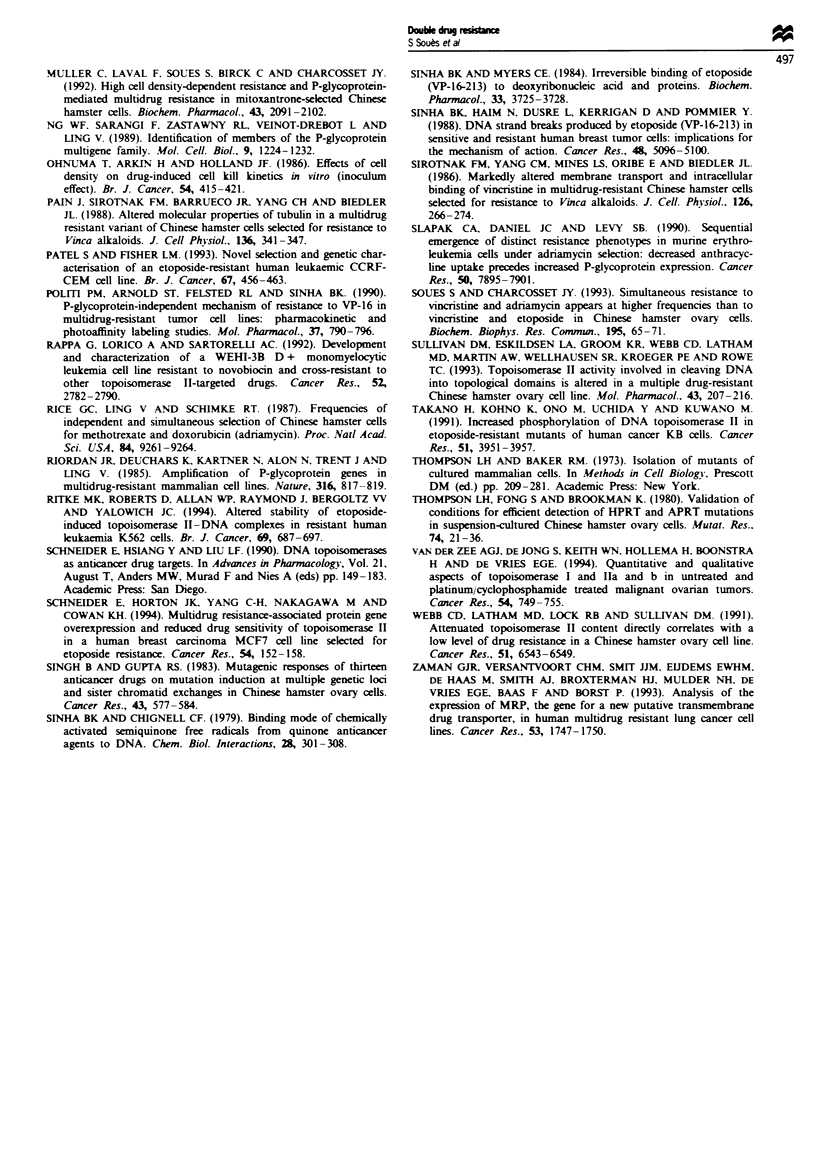# Mechanisms of resistance to combinations of vincristine, etoposide and doxorubicin in Chinese hamster ovary cells.

**DOI:** 10.1038/bjc.1995.99

**Published:** 1995-03

**Authors:** S. Souès, F. Laval, J. Y. Charcosset

**Affiliations:** Laboratoire de Pharmacologie et de Toxicologie Fondamentales, CNRS, Toulouse, France.

## Abstract

**Images:**


British Journal of Cancer (1995) 71, 489-497

© 1995 Stockton Press All rights reserved 0007-0920/95 $9.00

### Mechanisms of resistance to combinations of vincristine, etoposide and doxorubicin in Chinese hamster ovary cells

S Soués, F Laval and J-Y Charcosset

*Laboratoire de Pharmacologie et de Toxicologie Fondamentales, CNRS, 205 route de Narbonne, 31077 Toulouse Cédex, France.*

**Summary** We have isolated from Chinese hamster ovary cells, 30 sublines resistant to vincristine, doxorubicin or etoposide and 43 sublines evading treatment with a pair of these drugs. Isolated in one step and under low selective pressure, sublines were 3- to 25-fold more resistant to their selecting drug(s) than the parental cells. Possible P-glycoprotein-associated multidrug resistance was investigated through *pgp* gene copy number and mRNA expression level. DNA topoisomerase II alteration was evaluated from the ability of nuclear extracts to form cleavable complexes. Vincristine (all sublines) and doxorubicin (6/7 sublines) preferentially selected for *pgp* gene amplification and mRNA overexpression, whereas selection with etoposide resulted in a decrease of cleavable complex formation in 11 out of 13 sublines. A common *pgp* gene-mediated resistance was found in the 13 doxorubicin plus vincristine-selected sublines, whereas all but one of the 12 etoposide plus vincristine-resistant sublines displayed both *pgp* mRNA overexpression and decreased ability to form cleavable complexes. Among the 18 doxorubicin plus etoposide selected sublines, five exhibited a decreased ability to form cleavable complexes only, six exhibited *pgp* mRNA overexpression only and six exhibited both alterations. Overall, drug resistance could not be attributed to either mechanism in three of the 73 sublines. We conclude that under low selective pressure it is possible to find a combination of drugs which require simultaneous selection of more than one resistance mechanism; such cells emerge with very low frequency.

**Keywords**: drug resistance; combined alterations; multidrug resistance; DNA topoisomerase II; *pgp* genes

Drug resistance is, beside the poor selectivity of antitumoral agents, the main limitation of cancer chemotherapy (Goldie and Coldman, 1984). Regimens usually involve at least two drugs, but resistance often develops simultaneously to several agents. Typically, the frequency of resistance to combined drugs is much higher than the product of the monoresistant frequencies (Giulotto *et al.*, 1987; Rice *et al.*, 1987; Soués and Charcosset, 1993). Multiple immunities may result from a specific phenotype, characterised by DNA amplification. For instance, the high frequency of simultaneous resistance to methotrexate and *N*-phosphonacetyl-L-aspartate is due to gene amplification of both target enzymes: dihydrofolate-reductase and polypeptide having carbamyl phosphate synthetase, aspartate transcarbamylase and dihydroorotase activities (CAD) (Giulotto *et al.*, 1987). Similarly, concurrent treatment with methotrexate and doxorubicin (DOX) induces amplification of the dihydrofolate reductase and of the multidrug resistance-associated genes (Rice *et al.*, 1987). Multiple drug resistance (MDR) may also follow overexpression of the multidrug efflux pump P-glycoproteins (PGP), coded by three classes of genes (Georges *et al.*, 1990; Gottesman and Pastan, 1993). Transfection of hamster *pgp1* (class I genes) and of mouse *mdr1* gene, homologous to the hamster *pgp2* gene (class II genes), has been shown to induce the classical MDR phenotype (Gottesman and Pastan, 1993; Devine and Melera, 1994). Modification of DNA topoisomerase II, or a nuclear decrease in this enzyme, may also confer resistance to several of its poisons (Charcosset *et al.*, 1988; Fernandes *et al.*, 1990; Schneider *et al.*, 1990; Harker *et al.*, 1991; Hinds *et al.*, 1991; Rappa *et al.*, 1992), the phenotype being referred to as atypical MDR (at MDR) (Danks *et al.*, 1988).

Etoposide (ETO) is an epipodophyllotoxin derivative which selects cells with quantitative and/or qualitative alterations of DNA topoisomerase II (Ferguson *et al.*, 1988; Long *et al.*, 1991; Takano *et al.*, 1991; Webb *et al.*, 1991; Danks *et al.*, 1993; Patel and Fisher, 1993; Sullivan *et al.*, 1993; Ritke *et al.*, 1994). To date, only one cell line exhibited *mdr1* mRNA overexpression, without topoisomerase II modification, after selection by teniposide (Long *et al.*, 1991). Vincristine (VCR) is a vinca alkaloid which selects for modified tubulin, its target (Houghton *et al.*, 1985; Pain *et al.*, 1988), or for PGP overexpression (Georges *et al.*, 1990). Finally, DOX is a DNA intercalative drug, which selects for DNA topoisomerase II modification (De Jong *et al.*, 1990; McPherson *et al.*, 1993) as well as for the MDR phenotype (Georges *et al.*, 1990; Slapak *et al.*, 1990).

In previous studies, we reported that the frequencies of resistance to combinations of DOX, VCR and ETO suggest that both common and independent mechanisms of resistance can be selected in Chinese hamster ovary (CHO) cells (Soués and Charcosset, 1993). In this paper, we document that VCR and DOX preferentially select CHO cells with amplification and overexpression of the *pgp* genes, whereas DNA topoisomerase II alteration almost systematically accounts for ETO resistance. We also provide evidence that amplification of the *pgp* genes develops without DNA topoisomerase II modification after treatment with VCR plus DOX, whereas both *pgp* gene amplification and DNA topoisomerase II alteration occur in VCR plus ETO-selected sublines. When the selection is achieved with a combination of DOX plus ETO, drug resistance is ensured by either or both mechanisms. We conclude that cells selected with two drugs tend to exhibit a mechanism that allows them to resist both drugs simultaneously. Alternatively cells need two, independent, mechanisms of resistance.

### Materials and methods

### Cell lines and cell culture

The Chinese hamster ovary (CHO) cells AA8 (Thompson *et al.*, 1980) were used as parental cell line and grown as previously described (Muller *et al.*, 1992). Chinese hamster ovary cell line AuxB1 and its colchicine-resistant subline $CH^RC5$ (Kartner *et al.*, 1985) were kindly provided by Dr RM Baker (RPMI, Buffalo, NY, USA).

### Drugs

Aqueous solution of VCR (Oncovin) was purchased from Lilly France (Saint Cloud, France). DOX (Adriblastine) was

Correspondence: S Soués, Clinical Oncology and Radiotherapeutics Unit, MRC Centre, Hills Road, Cambridge CB2 2QH, UK
Received 1 August 1994; revised 3 November 1994; accepted 4 November 1994.

purchased from Laboratoire Roger Bellon (Neuilly-sur-Seine, France) and stored at −20°C after dissolution in water at 1 mg $ml^{-1}$. ETO was kindly provided by Drs HM Holava and JD Matiskella (Bristol Myers, Wallingford, CT, USA) or by Dr C Dubray (Laboratoires Sandoz, Rueil-Malmaison, France), and 10 mM aliquots in dimethylsulphoxide were stored at −20°C.

### *Selection of resistant cells and dose-dependent survival analysis*

Selection of AA8 cells ($10^5$ to $10^6$ cells in 100 mm Petri dishes) was achieved in 13–15 days in the continuous presence of the selecting drug concentration. Care was taken to avoid cell-to-cell contact for possible metabolic cooperation (Hooper and Subak-Sharpe, 1981). Colonies of various morphologies were isolated, and expanded in the presence of the selecting drug(s) for up to 2 months, before constitution of frozen stocks. Each selected subline is defined according to its parental cells (AA8), followed by the selecting drug(s) used (DOX, VCR and/or ETO) and a clone number (i.e. AA8/DOX + VCR.B6 stands for clone B6 selected from AA8 cells by DOX plus VCR).

Drug cytotoxicity was determined by colony formation assay after 6–8 days of continuous exposure to the drug, as previously (Muller *et al*., 1992). No inoculum effect was observed with either parental or selected cells (Ohnuma *et al*., 1986). Each cell line was characterised by the concentration of drug resulting in 10% survival (D10) and by its resistance index (RI), defined as the ratio of the D10 value for a particular subline over that for the parental cell line.

### *Northern and Southern blots*

RNA and DNA were extracted by the guanidine thiocyanate method, separated using a caesium chloride cushion (Chirgwin *et al*., 1979; Muller *et al*., 1992) and purified by phenol–chloroform extraction. Four micrograms of total RNA was resolved in 1.2% agarose gel containing 2.2 M formaldehyde, and Northern blots were performed as described by Dautry *et al*. (1988). The amount of rRNA loaded was quantified by ethidium bromide staining (Muller *et al*., 1992), and that of actin mRNA was probed, using a transcript of pBACT 5 plasmid containing the 600 bp *TagI*/*PstI* fragment of the mouse gene (Dautry *et al*., 1988), after labelling with [$^{32}$P]UTP. A 1.2 kb cDNA fragment encompassing from nucleotide 3051 to the poly-A tail of the human *mdr*1 gene (kindly provided by Dr T Tsuruo, Tokyo, Japan) was labelled with [α-$^{32}$P]dCTP, and used to probe the *pgp* hamster mRNA. The amount of *pgp* mRNA in each cell line was quantified by densitometry of the autoradiograms, using a double-beam microdensitometer (MKIIIC, Joyce, Loebl, UK), and averaged from a minimum of two RNA extractions. Ten micrograms of total cellular DNA was developed in 0.8% agarose gel after complete digestion by *Eco*RI. Southern blots were probed using the 1.2 kb human *mdr*1 cDNA insert, which hybridised to eight *Eco*RI fragments, covering the three *pgp* genes (Riordan *et al*., 1985; Ng *et al*., 1989; Muller *et al*., 1992). DNA and RNA from CHO $CH^RC5$ and AuxB1 cells were extracted, and analysed as positive and negative controls respectively.

### *Preparation of the nuclear extracts and DNA topoisomerase II assay*

Nuclear extracts were prepared according to Glisson *et al*. (1986) with minor modifications as described by Charcosset *et al*. (1988). Briefly, 4–6 × $10^7$ exponentially growing cells were trypsinised, washed with phosphate-buffered saline, and the nuclei were extracted at 4°C by Dounce homogenisation in a swelling buffer containing 0.3% Triton X-100 (v/v). Nuclei were further purified by centrifugation through a sucrose cushion, and the concentration of sodium chloride was raised to 0.35 M to extract DNA topoisomerase II. For the purpose of comparison, AA8 cells were processed systematically with resistant sublines. In preliminary experiments we determined that the protein content in nuclear extracts was directly proportional to the initial number of cells used for the extraction. Therefore, the total protein concentration was used to normalise the nuclear extracts.

Etoposide-stimulated DNA cleavage activity was assayed using the ability of the 0.35 M sodium chloride nuclear extract to linearise supercoiled pBR 322 DNA, as described by Charcosset *et al*. (1988). Samples were analysed by gel electrophoresis in 0.8% agarose gel in the presence of ethidium bromide and pictures were taken under UV light. The percentage of form III plasmid (linear) was estimated by scanning negative films. With all sublines, the DNA cleavage activity of the nuclear extracts increased with the concentration of ETO added and plateaued for concentrations higher than 64 μM. The percentages of cleavable complexes reported in this study correspond to that measured for a given subline, relative to that for the parental cell line, in the presence of a saturating concentration of ETO (128 μM).

### Results

### *Selection of AA8 colonies resistant to DOX, VCR and/or ETO*

We have isolated from CHO-AA8 cells, under low selective pressure and in a single step, 30 sublines resistant to either DOX, VCR or ETO, and 43 sublines resistant to combinations of these drugs. D10 values for DOX, VCR and ETO of the AA8 cells were 59 ± 2.6 nM, 23 ± 4.5 nM and 630 ± 70 nM respectively (mean ± s.d. of 4–10 determinations, each in triplicate). Selecting concentrations, frequency of surviving cells, number of colonies obtained and plating efficiencies are summarised in Table I. To select single drug-resistant colonies, we used 3 × D10, which induced a frequency of surviving cells of about $10^{-5}$ to $10^{-4}$. To obtain doubly resistant colonies, we combined 0.28 μM DOX with 0.05 μM VCR or 1.6 μM ETO (or VCR and ETO at the same concentrations). These combinations induced a frequency of surviving cells of $10^{-7}$ to $10^{-6}$. The frequency of surviving cells was 700-fold greater than the product of each single drug resistance frequency with the association DOX plus VCR, approximately equal to this product with VCR and ETO, and intermediate with DOX plus ETO (Soués and Charcosset, 1993).

The plating efficiency of each subline was determined in the absence as well as in the presence, of the selecting drug (Table I). Without drug, plating efficiencies were in the range

**Table I** Sensitivity of AA8 cells to DOX, VCR or ETO, selective conditions and plating efficiencies of sublines resistant to DOX, VCR or ETO or to a combination of these drugs

| *Subline* | *Selecting concentrations (μM)* DOX | VCR | ETO | *Selecting frequency* | *Number of colonies* | *Plating efficiency (%)*[a] No drug | DOX | VCR | ETO |
|---|---|---|---|---|---|---|---|---|---|
| AA8/DOX | 0.18 | – | – | $5 \times 10^{-5}$ | 7 | 42–57[b] | 30–46 | – | – |
| AA8/VCR | – | 0.07 | – | $2 \times 10^{-5}$ | 10 | 67–89 | – | 44–66 | – |
| AA8/ETO | – | – | 2.00 | $2 \times 10^{-4}$ | 13 | 44–88 | – | – | 34–51 |
| AA8/DOX + VCR | 0.28 | 0.05 | – | $2 \times 10^{-7}$ | 13 | 48–88 | 30–75 | 25–60 | – |
| AA8/VCR + ETO | – | 0.05 | 1.60 | $2 \times 10^{-6}$ | 12 | 38–82 | – | 32–72 | 30–73 |
| AA8/DOX + ETO | 0.28 | – | 1.60 | $8 \times 10^{-7}$ | 18 | 40–70 | 25[c]–61 | – | 26–63 |

[a]Plating efficiency was determined by colony-forming assay, under continuous exposure to the drug. [b]Range: each value is the mean of 2–5 determinations (each in triplicate). [c]The plating efficiencies of 2 of the 18 sublines were 4% and 6%.

of 40–90%. In the presence of the selecting drug, cells retained 51–91% of this plating efficiency. Thus most sublines effectively resisted their selecting drug (Thompson and Baker, 1973). However, sublines exhibited relatively low level of resistance to the selecting drug: RI values extended from 3- to 25-fold (Figures 1–6). In an attempt to identify possible PGP overexpression and/or topoisomerase II alteration, we characterised these sublines for *pgp* gene copy number, *pgp* mRNA overexpression and the ability of the nuclear extracts to form cleavable complexes in the presence of ETO.

### *Characterisation of the DOX-resistant sublines*

Among the DOX-selected sublines, six out of seven exhibited *pgp* gene amplification as well as *pgp* mRNA overexpression, whereas formation of cleavable complexes was similar to that of the parental cells (Figure 1). Amplification of the *pgp* genes appeared homogeneous in sublines AA8/DOX.A1, A3, A4 and A5 (i.e. the relative intensities of the *Eco*RI DNA fragments were similar to that of the AA8 cells). In contrast, in sublines AA8/DOX.A6 and B6, the most amplified *Eco*RI fragments were those corresponding to the *pgp*2 gene, whereas the bands of *pgp*3 gene were the least amplified. The level of mRNA overexpression was not related to the gene amplification pattern or level. For instance, sublines AA8/DOX.A1 and A6 had the highest level of mRNA detectable, but different *pgp* gene amplification pattern and level. In addition, there was no correlation between the sensitivity to DOX and the level of *pgp* gene amplification or mRNA overexpression: RI values were between 5 and 10 with all

**Figure 1** Characterisation of the DOX-resistant sublines. Top: resistance indexes to DOX of the selected sublines. Middle: Southern blot analysis. The amount of DNA loaded on the gel was 10 μg for the CHO cells and 3 μg for the CH$^R$C5 cells (used as positive control). The *Eco*RI fragments which hybridise to the human *mdr*1 probe are each attributed to one of the hamster *pgp* genes, according to their size in kilobases. Sublines A6 and B6 presented a preferred *pgp*2 amplification pattern. In the other sublines, the relative intensity of each band was similar to that of the AA8 parental cells. The lower panel indicates the fold increase in the *pgp* mRNA expression level (relative to the average obtained with the AA8 cells). There was no *pgp* gene amplification in subline A2, which was the only one to exhibit a decreased ability to form cleavable complexes (not shown).

**Figure 2** Characterisation of the VCR-resistant sublines. Top: resistance indexes to VCR. Middle: Southern blot analysis. Bottom: fold increase in *pgp* mRNA level (as in Figure 1). All sublines exhibited both *pgp* gene amplification and mRNA overexpression. Sublines B16, C13 and C14 exhibited a homogeneous *pgp* gene amplification pattern, the other sublines a specific *pgp*1 amplification. As expected with VCR-selected cells, no decrease in the ability to form cleavable complexes was detected (not shown).

sublines (Figure 1). In the seventh subline (AA8/DOX.A2), neither *pgp* gene amplification nor mRNA overexpression was detected, but *in vitro* drug-dependent DNA cleavage was only 50% that of the AA8 cells, suggesting a decrease in the ability to stabilise cleavable complexes. Topoisomerase II alteration, and *pgp* gene amplification can both confer cross-resistance to several drugs. Therefore we examined the resistance of four sublines to VCR and ETO. Sublines AA8/DOX.A4, A5 and A6, with *pgp* gene amplification, presented a cross-resistance to VCR and ETO (RI values were between 4 and 6, Table II). Subline AA8/DOX.A2, with a reduced ability to form cleavable complexes, retained sensitivity to VCR (RI value of 0.9), but was 3-fold cross-resistant to ETO.

Thus, DOX preferentially selects AA8 cells with *pgp* gene amplification, resulting in VCR and ETO cross-resistance. Modification of topoisomerase II (without *pgp* gene amplification) accounted for DOX resistance and ETO cross-resistance in one subline only.

*Characterisation of the VCR-resistant sublines*

Compared with the parental cells, *pgp* genes were amplified in all VCR-selected sublines, whereas *pgp* mRNA was moderately overexpressed (Figure 2). In sublines AA8/VCR.B16, C13 and C14, the relative intensities of the DNA fragments were similar to that of the parental cells, but in sublines AA8/VCR.A12, B11, B12, B14, C12 and C16, *pgp*1 gene was preferentially amplified. The *pgp*1 gene was particularly amplified in subline AA8/VCR.A11, but the intensity of the 4.2 kb fragment of *pgp*2 gene was also increased. Here the RI values were related to the level of *pgp*1 amplification. Sublines with preferential *pgp*1 gene amplification had RI values higher than 11, and when there was homogeneous amplification the RI was still higher for sublines with high *pgp*1 copy number. In fact, subline AA8/VCR.C14, with the lowest *pgp*1 amplification, had the lowest RI value (4.8). Three sublines with representative amplification pattern (AA8/VCR.B14, B16 and C13) exhibited cross-resistance to both DOX and ETO (RI values higher than 5.3, Table II), whereas subline AA8/VCR.C14 was both DOX and ETO sensitive (RI values of 1.1 and 1.5 respectively). It is unlikely that topoisomerase II modification had been co-selected with *pgp* amplification. Indeed, the capacity to form cleavable complexes was similar to that of the AA8 parental cells in sublines AA8/VCR.B14, B16, C12, C13 and C14.

Taken together, these data suggest that VCR typically selects AA8 cells with amplification and overexpression of *pgp* genes resulting in cross-resistance to DOX and ETO.

**Figure 3** Characterisation of the ETO-resistant sublines. Top: resistance indexes to ETO. Bottom: ability to form cleavable complexes (expressed as a percentage of that determined for the AA8 parental cells); n (for normal) indicates that no difference with the AA8 parental cells could be detected. All but two sublines (B4 and C3) had a reduced ability to form cleavable complexes. There was no detectable *pgp* gene amplification with any of the sublines (the amount of each *pgp* gene was similar to that of the parental cells), but subline C6 exhibited a slight increase in mRNA level (not shown).

*Characterisation of the ETO-resistant sublines*

A decreased ability to form cleavable complexes was observed in 11 out of the 13 ETO-resistant sublines, whereas none exhibited *pgp* gene amplification (Figure 3). The percentages of drug-stimulated DNA cleavage were 2- to 20-fold lower than that of the parental cells, and RI values varied between 3.5 and 19. Among these sublines, AA8/ETO.C6, with a high RI value (11.9), exhibited a slight *pgp* mRNA overexpression. Sublines AA8/ETO.B4 and C3 are potentially interesting, because neither *pgp* gene amplification, mRNA overexpression nor decreased cleavable complex formation could explain their high RI values (10.8 and 18.9 respectively). ETO and DOX are both topoisomerase II poisons, and VCR, while a tubulin binder, preferentially selects PGP-associated multidrug resistance. Possible cross-resistance to DOX or VCR of sublines AA8/ETO.A5, B1 and B2 was tested. Cross-resistance to DOX was only about

**Figure 4** Characterisation of the DOX plus VCR-resistant sublines. Top: resistance indexes to DOX (■) and to VCR (▨). Middle: Southern blot analysis. Bottom: fold increase in *pgp* mRNA level (as in Figure 1). All sublines exhibited a homogeneous *pgp* gene amplification pattern, except subline B3, which presents a preferred *pgp*2 amplification pattern. All sublines overexpressed to various extents the *pgp* mRNA. No alteration of the ability to form cleavable complexes was detected (not shown).

2-fold, and cells retained sensitivity to VCR (RI values were between 0.9 and 1.2; Table II).

Therefore, ETO preferentially selects AA8 cells through topoisomerase II modification. Yet, the highest RI values were obtained with a concomitant *pgp* gene overexpression in one subline, and through neither mechanism in two sublines.

*Characterisation of the DOX plus VCR-resistant sublines*

All the sublines selected with a combination of DOX and VCR exhibited *pgp* gene amplification and *pgp* mRNA overexpression, but, in contrast to the VCR-selected cells, there was no evidence of specific *pgp*1 amplification (Figure

**Figure 5** Characterisation of the ETO plus VCR-resistant sublines. Top: resistance indexes to ETO ( □ ) and to VCR ( ▨ ). Middle: Southern blot analysis. Bottom: fold increase in *pgp* mRNA level (as in Figure 1) and ability to form cleavable complexes (as in Figure 3). Seven sublines exhibited a homogeneous *pgp* gene amplification pattern (A3, B2, B3, C3, C6, F2 and G2), the other sublines (A6, B1, B4, E3 and F3) a preferred *pgp*2 amplification pattern. All sublines overexpressed to various extents the *pgp* mRNA, and all but subline E3 had a reduced ability to form cleavable complexes.

**Figure 6** Characterisation of the DOX plus ETO-resistant sublines. Top: resistance indexes to DOX ( ■ ) and to ETO ( □ ). Middle: Southern blot analysis and Northern blot analysis [total RNA (4 μg) was hybridised to the human *mdr*1 probe; the arrows indicate the position of 28S rRNA]. Bottom: fold increase in *pgp* mRNA level (as in Figure 1) and ability to form cleavable complexes (as in Figure 3). Sublines A5, C3, D5, E4, G5 and H6 exhibited *pgp* mRNA overexpression only, sublines A3, C5, D2, G1 and G4 exhibited a decreased ability to form cleavable complexes only and sublines A2, A4, B1, B4, C6 and G3 exhibited both alterations. In subline E5 neither *pgp* gene overexpression nor topoisomerase II alteration could account for the resistance to DOX and ETO.

**Table II** Resistance (bold) and cross-resistance to DOX, VCR or ETO of selected sublines

| | Resistance indexes to | | |
|---|---|---|---|
| *Resistant subline* | *DOX* | *VCR* | *ETO* |
| AA8/DOX.A2 | **8.1**[a] | 0.9 | 3 |
| AA8/DOX.A4 | **10.2** | 6.2 | 4 |
| AA8/DOX.A5 | **10.2** | 5.9 | 5.4 |
| AA8/DOX.B6 | **9.7** | 5.3 | 6.3 |
| AA8/VCR.B14 | 5.3 | **11.3** | 6 |
| AA8/VCR.B16 | 10.4 | **8.3** | 7 |
| AA8/VCR.C13 | 10.2 | **7.4** | 5.4 |
| AA8/VCR.C14 | 1.1 | **4.8** | 1.5 |
| AA8/ETO.A5 | 1.9 | 1.0 | **8.9** |
| AA8/ETO.B1 | 2.1 | 0.9 | **5.1** |
| AA8/ETO.B2 | 2.0 | 1.2 | **7.5** |

[a]*P*-values for comparison of the mean D10 subline and D10 AA8 were 0.0012–0.032 (significant differences) when RIs were equal or above 1.9 and 0.084–0.46 (not significant differences) when RIs were between 0.9 and 1.5.

4). Compared with the parental cells, the relative intensities of the DNA fragments were similar in all but one subline. Interestingly, the pattern of the 13th subline (AA8/DOX + VCR.B3) was similar to that of the DOX-selected cells AA8/DOX.A6 and B6: *pgp2* fragments were the most intense and the *pgp3* gene was less amplified than the *pgp1* gene. Subline AA8/DOX + VCR.B3 also had the highest level of *pgp* mRNA overexpression among the doubly resistant cells. In spite of the higher selective drug concentration, the resistance to DOX of the DOX plus VCR-selected cells was similar to that obtained with the DOX-selected sublines. In contrast, possibly because of the lack of specific *pgp1* gene amplification, RI values to VCR followed the selective drug concentration: values obtained with a lower selective drug concentration in the doubly resistant sublines were lower than that observed with the VCR-selected cells. mRNA overexpression and *pgp* gene amplification are potentially sufficient to evade DOX plus VCR treatment, but topoisomerase II modification could also induce resistance to DOX. Three sublines (AA8/DOX + VCR.B3, C1 and D1) were tested for their ability to form cleavable complexes, but all extracts had the same ability as the AA8 parental extract to induce cleavable complex formation.

Taken together, these results suggest that, rather than DNA topoisomerase II alteration, increased *pgp* gene copy number and mRNA overexpresssion account for the double resistance to VCR and DOX.

### *Characterisation of the VCR plus ETO-resistant sublines*

All but one of the 12 sublines which resisted the combination of ETO plus VCR had a reduced ability (up to 30%) to form cleavable complexes (Figure 5). These 12 sublines also exhibited *pgp* gene amplification as well as *pgp* mRNA overexpression. No subline presented a specific *pgp1* amplification pattern. Compared with the parental cells, the relative intensities of the DNA fragments were similar in seven sublines (AA8/ETO + VCR.A3, B2, B3, C3, C6, F2 and G2). The five other sublines (AA8/ETO + VCR.A6, B1, B4, E3 and F3) exhibited a pattern similar to that of AA8/DOX + VCR.B3, AA8/DOX.A6 and B6 cells (i.e. the *pgp2* gene was more amplified than the *pgp1* gene, itself more amplified than the *pgp3* gene). As for AA8/DOX + VCR cells, RI values obtained with VCR were lower (mean value of 6) than for the cells selected with VCR only (mean value of 11.6).

Therefore, two, independent mechanisms of resistance seem to be required to ensure survival to a combination of ETO and VCR.

### *Characterisation of the DOX plus ETO-resistant sublines*

All combinations of resistance mechanisms were found within the 18 DOX plus ETO-selected sublines (Figure 6). Amplification of the *pgp* genes ranged from very high to non-detectable and ability to form cleavable complexes ranged from 10% to indistinguishable from that of the parental cells. Five sublines exhibited only a decreased ability to form cleavable complexes (AA8/DOX + ETO.A3, C5, D2, G1 and G4), six displayed *pgp* mRNA overexpression only (AA8/DOX + ETO.A5, C3, D5, E4, G5 and H6), whereas both mechanisms were present in sublines AA8/DOX + ETO.A2, A4, B1, B4, C6 and G3. In one subline (AA8/DOX + ETO.E5), neither *pgp* gene amplification nor decreased ability to form cleavable complexes could account for the resistance. As for most of the DOX-selected sublines, and for all but one of the DOX plus VCR-selected sublines, when *pgp* gene amplification was observed the relative intensities of the *Eco*RI fragments were similar to that of the AA8 parental cells. Various levels of *pgp* mRNA expression were observed, but, as previously, no correlation between *pgp* gene amplification and mRNA expression could be drawn. Most high RI values to ETO were among the cells presenting a decreased ability to form cleavable complexes, but a 30% decrease in the ability to stabilise cleavable complexes was associated with both the greatest RI value to ETO (25 in subline AA8/DOX + ETO.G4), and the RI value of 11 in subline AA8/DOX + ETO.A3. Furthermore, subline AA8/DOX + ETO.H6, with *pgp* amplification only, had a RI value to ETO of 24.

Our results suggest that either of the two mechanisms, *pgp* mRNA overexpression or decreased ability to form the cleavable complexes, can induce resistance to DOX plus ETO.

### Discussion

In this study, we verify that DOX, VCR and ETO preferentially select one mechanism of resistance in CHO-AA8 cells, and we investigate whether there is common or independent mechanisms of resistance to combinations of these antitumoral drugs. We show that DOX and VCR preferentially select amplification of the *pgp* gene-associated multidrug resistance, whereas ETO resistance results primarily from topoisomerase II alteration. We unveil common resistance mechanisms to combination of these drugs, except for the VCR plus ETO association, which requires selection of two independent mechanisms.

Clinically used concentrations of antitumoral drugs induce emergence of resistant cells. We observed, however, that a low selective pressure results in a moderate level of resistance in CHO-AA8 sublines. The alterations conferring resistance to the antitumoral agents could not result from a mutagenic response owing to a long exposure to drug or from pretreatment with a mutagen (Singh and Gupta, 1983). Therefore, it is likely that the selection resulted from a threshold effect: there were pre-existing mutants which emerged from the parental population. Consistent with our observations, a low concentration of vinblastine or colchicine with human melanoma cells (Lemontt *et al.*, 1988), or of DOX with murine erythroleukaemia cells (Slapak *et al.*, 1990) also induce low levels of resistance.

Overexpression of the *pgp* genes occurred in most CHO-AA8 sublines selected with VCR or DOX; only one of the DOX-resistant sublines exhibited a modified ability to form cleavable complexes. Typically, cells selected with DOX (or VCR) and featuring *pgp* gene amplification were cross-resistant to ETO and VCR (or DOX). The ETO-selected cells exhibited primarily a modified drug-stimulated ability to stabilise cleavable complexes; this includes the only subline which slightly overexpressed *pgp* mRNA. Cells selected with ETO (or DOX) and featuring topoisomerase II alteration were cross-resistant to DOX (or ETO), but retained their sensitivity to VCR. Consistent with our data, VCR selects for *pgp* overexpression, and ETO selects for topoisomerase II alteration, whereas both mechanisms of resistance may apply with DOX. The three drugs are potentially recognised by PGP, but only ETO and DOX can stabilise the complexes between DNA and topoisomerase II (Liu, 1989; Georges *et al.*, 1990; Corbett *et al.*, 1993; Gottesman and Pastan, 1993). Alterations of topoisomerase II, without overexpression of PGP, have been observed in P388 murine leukaemia cells and human small-cell lung carcinoma cells selected with DOX (De Jong *et al.*, 1990; McPherson *et al.*, 1993). In addition, very few cell lines exhibited the classical MDR phenotype after selection with ETO or teniposide (Long *et al.*, 1991; Hosking *et al.*, 1994). There was no correlation between *pgp* gene amplification or mRNA overexpression and level of resistance. Regulation of the transcription and/or mRNA stabilisation may control PGP expression. Overexpression of PGP without increase in *pgp* mRNA has been observed in tumour cells resistant to vinca alkaloids (Bradley *et al.*, 1989; Hill *et al.*, 1990; Biedler, 1994). Resistance to VCR could result from tubulin alteration (Houghton *et al.*, 1985; Sirotnak *et al.*, 1986; Pain *et al.*, 1988). Such a mechanism possibly accounts for two of the VCR-resistant sublines exhibiting only a slight increase in *pgp* mRNA. Alternatively, point mutations in PGP could affect the transporter affinity for a particular drug (Gottesman and Pastan, 1993). Finally, specific drug immunity may be associated with a particular *pgp* gene (Georges *et al.*, 1990; Gottesman and Pastan, 1993). Three patterns of *pgp* gene amplification were established in the CHO-AA8 cells. The most common pattern was a general amplification of all three *pgp*1, *pgp*2 and *pgp*3 genes. A specific *pgp*1 amplification pattern was found in the VCR-selected cells, and a preferential amplification of *pgp*2 gene characterised some of the DOX-selected cells. In sublines displaying a general amplification pattern, the RIs to DOX were higher than those to VCR, and the resistance to ETO the lowest. In sublines with specific *pgp*1 amplification, the highest RIs were to VCR, and a relationship seemed to link the extent of *pgp*1 amplification to the level of VCR resistance. Similarly, a preferred *pgp*2 amplification may improve the resistance to DOX. We did not find a specific *pgp*3 gene amplification, but transfection of the human *mdr*3 gene (homologous to the hamster *pgp*3 gene) failed to induce the MDR phenotype in RBO melanoma cells (Gottesman and Pastan, 1993). While DOX and ETO are both topoisomerase II poisons, they appear to select preferentially different mechanisms of resistance. Topoisomerase II modifications obtained through DOX selection were far less frequent than those emerging through ETO selection. Actually, the unique DOX-selected subline exhibiting topoisomerase II alteration was only 3-fold cross-resistant to ETO. Conversely, the ETO-selected sublines which exhibited an impaired ability to form cleavable complexes were at most 2-fold cross-resistant to DOX. However, the DOX- (or VCR) resistant sublines (overexpressing the *pgp* genes) were cross-resistant to ETO, suggesting that *pgp* gene overexpression may have been sufficient to confer ETO resistance. Again, independently from the *pgp* gene amplification pattern, cross-resistance to ETO was lower than resistance to DOX (or VCR). Consistent with our data, PGP does not recognise ETO as efficiently as DOX (Politi *et al.*, 1990; Long *et al.*, 1991). While Giaccone *et al.* (1992) found a direct relationship between sensitivity to topoisomerase II poisons and expression of topoisomerase II, the sensitivity to ETO or teniposide does not always correlate with reduction in topoisomerase II level and/or activity (Danks *et al.*, 1988; Ferguson *et al.*, 1988; Matsuo *et al.*, 1990; Ritke *et al.*, 1994). In our study, cleavable complex formation was not directly related to the level of the drug resistance. It has been suggested that 0.35 M sodium chloride may not fully extract the topoisomerase II. Nevertheless, different mutations in topoisomerase II, which would induce various sensitivity to the selecting drug, may also reconcile these observations (Bugg *et al.*, 1991; Danks *et al.*, 1993). Cleavable complex formation accounts for both qualitative and quantitative modifications of either topoisomerase II isoform $\alpha$ or $\beta$ (Drake *et al.*, 1989; Van der Zee *et al.*, 1994). In addition, other cellular alterations may confer resistance to ETO. Two of the ETO-selected sublines exhibited neither *pgp* RNA overexpression nor reduced drug-stimulated DNA cleavage. Possible alternative mechanisms of resistance include overexpression of another multidrug transporter associated with the MDR phenotype (Grant *et al.*, 1994; Schneider *et al.*, 1994), inducible P450 dependent drug-metabolising activity (Sinha *et al.*, 1988) and alteration of an enzyme involved in the glutathione metabolism (Sinha and Myers, 1984; Haim *et al.*, 1987).

With all the DOX plus VCR-resistant sublines, there was a common mechanism of resistance, which corresponded to the one preferentially selected by each drug alone. Simultaneous resistance to both drugs may have resulted from overexpression of a single PGP (which would recognise both drugs). Alternatively, concurrent amplification of distinct *pgp* genes, coding for different PGP, may account for the double drug resistance (Ng *et al.*, 1989; Gottesman and Pastan, 1993). While the general *pgp* gene amplification observed supports the latter hypothesis, both are in agreement with the high frequency of cells surviving DOX plus VCR treatment, which is similar to the frequency resulting from selection with DOX or VCR alone (Soués and Charcosset, 1993). In contrast, the frequency observed with the ETO plus VCR-selected cells was rather low, suggesting that two mutational events may be necessary to ensure simultaneous resistance (Soués and Charcosset, 1993). Indeed, we observed, in all but one of the ETO plus VCR-selected sublines, both *pgp* mRNA overexpression and decreased ability to form cleavable complexes. While cells selected with ETO retained sensitivity to VCR, cells selected with VCR alone were cross-resistant to ETO. Two mechanisms of resistance were selected, yet resistance to VCR alone alleviated the sensitivity to ETO. It is conceivable that the relatively low cross-resistance to ETO in cells selected with VCR alone rationalises this apparent contradiction: the need for a higher resistance to ETO would exclude PGP as a major source of protection in the doubly selected sublines. With the DOX plus ETO-selected cells, either one or both mechanisms were observed. Here, each mechanism could account for the double resistance: *pgp* overexpression (preferentially selected by DOX) procured cross-resistance to ETO and topoisomerase II alteration (almost systematically selected by ETO) ascertained the resistance of one of the DOX-selected sublines. PGP overexpression seemed, however, to be required for high DOX resistance, and topoisomerase II modification was associated with high resistance to ETO. This observation is compatible with the low cross-resistance to DOX of the ETO-selected sublines and with the moderate cross-resistance to ETO of the DOX-resistant cells. DOX plus ETO combination selected a heterogeneous population with either or both common mechanisms. The selection of single and double mutants probably accounted for the intermediate frequency of resistant cells observed: higher than the product of single resistance frequencies (as with the VCR plus ETO-resistant cells), but lower than either of the single resistance frequencies, as with the VCR plus DOX-selected cells (Soués and Charcosset, 1993). Finally, in one of the ETO plus DOX-resistant sublines, neither *pgp* overexpression nor topoisomerase II alteration was detected. In addition, a high resistance to ETO was observed in some of the doubly resistant sublines, despite an unchanged ability to form cleavable complexes. While an increased level of PGP without mRNA overexpression cannot be ruled out (Bradley *et al.*, 1989; Hill *et al.*, 1990), other modifications, as above, may have conferred resistance to ETO (Sinha and Myers, 1984; Haim *et al.*, 1987; Sinha *et al.*, 1988; Schneider *et al.*, 1994) and DOX (Sinha and Chignell, 1979; Berlin and Haseltine, 1981; Zaman *et al.*, 1993; Barrand *et al.*, 1994). Resistance to both drugs could also result from alteration in the lethal processing of the cleavable complexes (Schneider *et al.*, 1990; Glisson *et al.*, 1992).

**Abbreviations:** PGP, P-glycoprotein; MDR, multidrug resistance; *mdr*, MDR-associated genes; *pgp*, MDR-associated genes in hamster species; VCR, vincristine; ETO, etoposide; DOX, doxorubicin (Adriblastine); RI, resistance index; D10, 10% survival dose; CAD, polypeptide having carbaymyl phosphate synthetase, aspartate transcarbamylase and dihydroorotase activities.

**Acknowledgements**

This work was supported by the Centre National de la Recherche Scientifique, the Association pour la Recherche contre le Cancer, and the Ligue Nationale Française contre le Cancer. The authors wish to thank Dr B Le Bonniec, from the Department of Haematology, for his invaluable help in the preparation of this manuscript and Dr R Hoffman from the MRC centre in Cambridge for his critical comments.

**References**

BARRAND MA, HEPPEL-PARTON A, WRIGHT KA, RABBITTS PH AND TWENTYMAN PR. (1994). A 190-kilodalton protein overexpresssed in non-P-glycoprotein-containing multidrug-resistant cells and its relationship to the MRP gene. *J. Natl Cancer Inst.*, **86**, 110–117.

BERLIN V AND HASELTINE WA. (1981). Reduction of adriamycin to a semiquinone free radical by NADPH cytochrome P-450 reductase produces DNA cleavage in a reaction mediated by molecular oxygen. *J. Biol. Chem.*, **256**, 4747–4756.

BIEDLER JL. (1994). Drug resistance: genotype versus phenotype – thirty-second G.H.A. Clowes Memorial Award lecture. *Cancer Res.*, **54**, 666–678.

BRADLEY G, NAIK M AND LING V. (1989). P-glycoprotein in multidrug-resistant human ovarian carcinoma cell lines. *Cancer Res.*, **49**, 2790–2796.

BUGG BY, DANKS MK, BECK WT AND SUTTLE DP. (1991). Expression of a mutant DNA topoisomerase II in CCRF-CEM human leukemic cells selected for resistance to teniposide. *Proc. Natl Acad. Sci. USA*, **88**, 7654–7658.

CHARCOSSET JY, SAUCIER JM AND JACQUEMIN-SABLON A. (1988). Reduced DNA topoisomerase II activity and drug-stimulated DNA cleavage in 9-hydroxyellipticine resistant cells. *Biochem. Pharmacol.*, **37**, 2145–2149.

CHIRGWIN JM, PRZYBYLA AE, McDONALD RJ AND RUTTER WJ. (1979). Isolation of biologically active ribonucleic acid from sources enriched in ribonuclease. *Biochemistry*, **18**, 5294–5299.

CORBETT AH, HONG D AND OSHEROFF N. (1993). Exploiting mechanistic differences between drug classes to define functional drug interaction domains on topoisomerase II. *J. Biol. Chem.*, **268**, 14394–14398.

DANKS MK, SCHMIDT CA, CIRTAIN MC, SUTTLE DP AND BECK WT. (1988). Altered catalytic activity of and DNA cleavage by DNA topoisomerase II from human leukemic cells selected for resistance to VM-26. *Biochemistry*, **27**, 8861–8869.

DANKS MK, WARMOTH MR, FRICHE E, GRANZEN B, BUGG BY, HARKER WG, ZWELLING LA, FUTSCHER BW, SUTTLE DP AND BECK WT. (1993). Single-strand conformational polymorphism analysis of the Mr 170,000 isozyme of DNA topoisomerase II in human tumor cells. *Cancer Res.*, **53**, 1373–1379.

DAUTRY F, WEIL D, YU J AND DAUTRY-VARSAT A. (1988). Regulation of *pim* and *myb* mRNA accumulation by interleukin 2 and interleukin 3 in murine hematopoietic cell lines. *J. Biol. Chem.*, **263**, 17615–17620.

DE JONG S, ZIJLSTRA JG, DE VRIES EG AND MULDER NH. (1990). Reduced DNA topoisomerase II activity and drug-induced DNA cleavage activity in an adriamycin-resistant human small cell lung carcinoma cell line. *Cancer Res.*, **50**, 304–309.

DEVINE SE AND MELERA PW. (1994). Functional studies with a full-length P-glycoprotein cDNA encoded by the hamster *pgp*1 gene. *Cancer Chemother. Pharmacol.*, **33**, 465–471.

DRAKE FH, HOFMAN GA, BARTUS HF, MATTERN MR, CROOKE ST AND MIRABELLI CK. (1989). Biochemical and pharmacological properties of p170 and p180 forms of topoisomerase II. *Biochemistry*, **28**, 8154–8160.

FERGUSON PJ, FISHER MH, STEPHENSON J, LI D-H, ZHOU B-S AND CHENG Y-C. (1988). Combined modalities of resistance in etoposide-resistant human KB cell lines. *Cancer Res.*, **48**, 5956–5964.

FERNANDES DJ, DANKS MK AND BECK WT. (1990). Decreased nuclear matrix DNA topoisomerase II in human leukemia cells resistant to VM-26 and m-AMSA. *Biochemistry*, **29**, 4235–4241.

GEORGES E, SHAROM FJ AND LING V. (1990). Multidrug resistance and chemosensitization: therapeutic implications for cancer chemotherapy. *Adv. Pharmacol.*, **21**, 185–220.

GIACCONE G, GAZDAR AF, BECK H, ZUNINO F AND CAPRANICO G. (1992). Multidrug sensitivity phenotype of human lung cancer cells associated with topoisomerase II expression. *Cancer Res.*, **52**, 1666–1674.

GIULOTTO E, KNIGHTS C AND STARK GR. (1987). Hamster cells with increased rates of DNA amplification, a new phenotype. *Cell*, **48**, 837–845.

GLISSON BS, GUPTA R, SMALLWOOD-KENTRO S AND ROSS WE. (1986). Characterization of acquired epipodophyllotoxin resistance in a Chinese hamster ovary cell line: loss of drug stimulated DNA cleavage activity. *Cancer Res.*, **46**, 1934–1939.

GLISSON BS, KILLARY AM, MERTA P, ROSS WE, SICILIANO J AND SICILLIANO MJ. (1992). Dissociation of cytotoxicity and cleavage activity induced by topoisomerase II-reactive intercalating agents in hamster–human somatic cell hybrids. *Cancer Chemother. Pharmacol.*, **31**, 131–138.

GOLDIE JH AND COLDMAN AJ. (1984). The genetic origin of drug resistance in neoplasms: implications for systemic therapy. *Cancer Res.*, **44**, 3643–3653.

GOTTESMAN MM AND PASTAN I. (1993). Biochemistry of multidrug resistance mediated by the multidrug transporter. *Annu. Rev. Biochem.*, **62**, 385–427.

GRANT CE, VALDIMARSSON G, HIPFNER DR, ALMQUIST KC, COLE SPC AND DEELEY RG. (1994). Overexpression of multidrug resistance-associated protein (MRP) increases resistance to natural product drugs. *Cancer Res.*, **54**, 357–361.

HAIM N, NEMEC J, ROMAN J AND SINHA BK. (1987). Peroxidase-catalysed metabolism of etoposide (VP-16-213) and covalent binding of reactive intermediates to cellular macromolecules. *Cancer Res.*, **47**, 5835–5840.

HARKER WG, SLADE DL, DRAKE FH AND PARR RL. (1991). Mitoxantrone resistance in HL-60 leukemia cells: reduced nuclear topoisomerase II catalytic activity and drug-induced DNA cleavage in association with reduced expression of the topoisomerase II$\beta$ isoform. *Biochemistry*, **30**, 9953–9961.

HILL BT, DEUCHARS K, HOSKING LK, LING V AND WHELAN RDH. (1990). Overexpression of P-glycoprotein in mammalian tumor cell lines after fractionated X irradiation in vitro. *J. Natl Cancer Inst.*, **82**, 607–612.

HINDS M, DEISSEROTH K, MAYES J, ALTSCHULER E, JANSEN R, LEDLEY FD AND ZWELLING LA. (1991). Identification of a point mutation in the topoisomerase II gene from a human leukemia cell line containing an amsacrine-resistant form of topoisomerase II. *Cancer Res.*, **51**, 4729–4731.

HOOPER ML AND SUBAK-SHARPE JH. (1981). Metabolic cooperation between cells. *Int. Rev. Cytol.*, **69**, 45–104.

HOSKING LK, WHELAN RDH, SHELLARD SA, DAVIES SL, HICKSON ID, DANKS MK AND HILL BT. (1994). Multiple mechanisms of resistance in a series of human testicular teratoma cell lines selected for increasing resistance to etoposide. *Int. J. Cancer*, **57**, 259–267.

HOUGHTON JA, HOUGHTON PJ, HAZELTON BJ AND DOUGLASS EC. (1985). *In situ* selection of a human rhabdomyosarcoma resistant to vincristine with altered $\beta$-tubulins. *Cancer Res.*, **45**, 2706–2712.

KARTNER N, EVERNDEN-PORELLE D, BRADLEY G AND LING V. (1985). Detection of P-glycoprotein in multidrug-resistant cell lines by monoclonal antibodies. *Nature*, **316**, 820–822.

LEMONTT JF, AZZARIA M AND GROS P. (1988). Increased *mdr* gene expression and decreased drug accumulation in multidrug-resistant human melanoma cells. *Cancer Res.*, **48**, 6348–6353.

LIU LF. (1989). DNA topoisomerase poisons as antitumor drugs. *Annu. Rev. Biochem.*, **58**, 351–375.

LONG BH, WANG L, LORICO A, WANG RC, BRATTAIN MG AND CASAZZA AM. (1991). Mechanisms of resistance to etoposide and teniposide in acquired resistant human colon and lung carcinoma cell lines. *Cancer Res.*, **51**, 5275–5284.

McPHERSON JP, BROWN GA AND GOLDENBERG GJ. (1993). Characterization of a DNA topoisomerase IIa gene rearrangement in adriamycin-resistant P388 leukemia: expression of a fusion messenger RNA transcript encoding topoisomerase IIa and the retinoic acid receptor a locus. *Cancer Res.*, **53**, 5885–5889.

MATSUO K, KOHNO K, TAKANO H, SATO S, KIUE A AND KUWANO M. (1990). Reduction of drug accumulation and DNA topoisomerase II activity in acquired teniposide-resistant human cancer KB cell lines. *Cancer Res.*, **50**, 5819–5824.

MULLER C, LAVAL F, SOUES S, BIRCK C AND CHARCOSSET JY. (1992). High cell density-dependent resistance and P-glycoprotein-mediated multidrug resistance in mitoxantrone-selected Chinese hamster cells. *Biochem. Pharmacol.*, **43,** 2091–2102.

NG WF, SARANGI F, ZASTAWNY RL, VEINOT-DREBOT L AND LING V. (1989). Identification of members of the P-glycoprotein multigene family. *Mol. Cell. Biol.*, **9,** 1224–1232.

OHNUMA T, ARKIN H AND HOLLAND JF. (1986). Effects of cell density on drug-induced cell kill kinetics *in vitro* (inoculum effect). *Br. J. Cancer*, **54,** 415–421.

PAIN J, SIROTNAK FM, BARRUECO JR, YANG CH AND BIEDLER JL. (1988). Altered molecular properties of tubulin in a multidrug resistant variant of Chinese hamster cells selected for resistance to *Vinca* alkaloids. *J. Cell Physiol.*, **136,** 341–347.

PATEL S AND FISHER LM. (1993). Novel selection and genetic characterisation of an etoposide-resistant human leukaemic CCRF-CEM cell line. *Br. J. Cancer*, **67,** 456–463.

POLITI PM, ARNOLD ST, FELSTED RL AND SINHA BK. (1990). P-glycoprotein-independent mechanism of resistance to VP-16 in multidrug-resistant tumor cell lines: pharmacokinetic and photoaffinity labeling studies. *Mol. Pharmacol.*, **37,** 790–796.

RAPPA G, LORICO A AND SARTORELLI AC. (1992). Development and characterization of a WEHI-3B D+ monomyelocytic leukemia cell line resistant to novobiocin and cross-resistant to other topoisomerase II-targeted drugs. *Cancer Res.*, **52,** 2782–2790.

RICE GC, LING V AND SCHIMKE RT. (1987). Frequencies of independent and simultaneous selection of Chinese hamster cells for methotrexate and doxorubicin (adriamycin). *Proc. Natl Acad. Sci. USA*, **84,** 9261–9264.

RIORDAN JR, DEUCHARS K, KARTNER N, ALON N, TRENT J AND LING V. (1985). Amplification of P-glycoprotein genes in multidrug-resistant mammalian cell lines. *Nature*, **316,** 817–819.

RITKE MK, ROBERTS D, ALLAN WP, RAYMOND J, BERGOLTZ VV AND YALOWICH JC. (1994). Altered stability of etoposide-induced topoisomerase II–DNA complexes in resistant human leukaemia K562 cells. *Br. J. Cancer*, **69,** 687–697.

SCHNEIDER E, HSIANG Y AND LIU LF. (1990). DNA topoisomerases as anticancer drug targets. In *Advances in Pharmacology*, Vol. 21, August T, Anders MW, Murad F and Nies A (eds) pp. 149–183. Academic Press: San Diego.

SCHNEIDER E, HORTON JK, YANG C-H, NAKAGAWA M AND COWAN KH. (1994). Multidrug resistance-associated protein gene overexpression and reduced drug sensitivity of topoisomerase II in a human breast carcinoma MCF7 cell line selected for etoposide resistance. *Cancer Res.*, **54,** 152–158.

SINGH B AND GUPTA RS. (1983). Mutagenic responses of thirteen anticancer drugs on mutation induction at multiple genetic loci and sister chromatid exchanges in Chinese hamster ovary cells. *Cancer Res.*, **43,** 577–584.

SINHA BK AND CHIGNELL CF. (1979). Binding mode of chemically activated semiquinone free radicals from quinone anticancer agents to DNA. *Chem. Biol. Interactions*, **28,** 301–308.

SINHA BK AND MYERS CE. (1984). Irreversible binding of etoposide (VP-16-213) to deoxyribonucleic acid and proteins. *Biochem. Pharmacol.*, **33,** 3725–3728.

SINHA BK, HAIM N, DUSRE L, KERRIGAN D AND POMMIER Y. (1988). DNA strand breaks produced by etoposide (VP-16-213) in sensitive and resistant human breast tumor cells: implications for the mechanism of action. *Cancer Res.*, **48,** 5096–5100.

SIROTNAK FM, YANG CM, MINES LS, ORIBE E AND BIEDLER JL. (1986). Markedly altered membrane transport and intracellular binding of vincristine in multidrug-resistant Chinese hamster cells selected for resistance to *Vinca* alkaloids. *J. Cell. Physiol.*, **126,** 266–274.

SLAPAK CA, DANIEL JC AND LEVY SB. (1990). Sequential emergence of distinct resistance phenotypes in murine erythroleukemia cells under adriamycin selection: decreased anthracycline uptake precedes increased P-glycoprotein expression. *Cancer Res.*, **50,** 7895–7901.

SOUES S AND CHARCOSSET JY. (1993). Simultaneous resistance to vincristine and adriamycin appears at higher frequencies than to vincristine and etoposide in Chinese hamster ovary cells. *Biochem. Biophys. Res. Commun.*, **195,** 65–71.

SULLIVAN DM, ESKILDSEN LA, GROOM KR, WEBB CD, LATHAM MD, MARTIN AW, WELLHAUSEN SR, KROEGER PE AND ROWE TC. (1993). Topoisomerase II activity involved in cleaving DNA into topological domains is altered in a multiple drug-resistant Chinese hamster ovary cell line. *Mol. Pharmacol.*, **43,** 207–216.

TAKANO H, KOHNO K, ONO M, UCHIDA Y AND KUWANO M. (1991). Increased phosphorylation of DNA topoisomerase II in etoposide-resistant mutants of human cancer KB cells. *Cancer Res.*, **51,** 3951–3957.

THOMPSON LH AND BAKER RM. (1973). Isolation of mutants of cultured mammalian cells. In *Methods in Cell Biology*, Prescott DM (ed.) pp. 209–281. Academic Press: New York.

THOMPSON LH, FONG S AND BROOKMAN K. (1980). Validation of conditions for efficient detection of HPRT and APRT mutations in suspension-cultured Chinese hamster ovary cells. *Mutat. Res.*, **74,** 21–36.

VAN DER ZEE AGJ, DE JONG S, KEITH WN, HOLLEMA H, BOONSTRA H AND DE VRIES EGE. (1994). Quantitative and qualitative aspects of topoisomerase I and IIa and b in untreated and platinum/cyclophosphamide treated malignant ovarian tumors. *Cancer Res.*, **54,** 749–755.

WEBB CD, LATHAM MD, LOCK RB AND SULLIVAN DM. (1991). Attenuated topoisomerase II content directly correlates with a low level of drug resistance in a Chinese hamster ovary cell line. *Cancer Res.*, **51,** 6543–6549.

ZAMAN GJR, VERSANTVOORT CHM, SMIT JJM, EIJDEMS EWHM, DE HAAS M, SMITH AJ, BROXTERMAN HJ, MULDER NH, DE VRIES EGE, BAAS F AND BORST P. (1993). Analysis of the expression of MRP, the gene for a new putative transmembrane drug transporter, in human multidrug resistant lung cancer cell lines. *Cancer Res.*, **53,** 1747–1750.